# Preference at First Sight: Effects of Shape and Font Qualities on Evaluation of Object-Word Pairs

**DOI:** 10.3390/vision6020022

**Published:** 2022-04-12

**Authors:** Olivia S. Cheung, Oliver Heyn, Tobiasz Trawiński

**Affiliations:** Department of Psychology, New York University Abu Dhabi, Abu Dhabi 129188, United Arab Emirates; oliver.heyn@nyu.edu (O.H.); trawint@hope.ac.uk (T.T.)

**Keywords:** preference, category, eye movement, shape, font

## Abstract

Subjective preferences for visual qualities of shapes and fonts have been separately reported. Such preferences are often similarly attributed to factors such as aesthetic impressions, attributed meaning from the visual properties, or processing fluency. Because shapes and fonts were rarely studied together, we investigated whether these qualities had a similar impact on preference judgment of object-word pairs. Each pair consisted of an abstract object with either preferred or disliked shape qualities and a pseudoword with either preferred or disliked font qualities. We found that only shape qualities, but not font qualities, influenced preference ratings of the object-word pairs, with higher preferences for pairs with preferred than disliked shapes. Moreover, eye movement results indicated that while participants fixated the word before the object, their prolonged fixation on the object when first attending to it might have contributed to the preference ratings. Nonetheless, other measures, including response times, total fixation numbers, and total dwell time, showed different patterns for shape and font qualities, revealing that participants attended more to objects with preferred than disliked shapes, and to words with disliked than preferred fonts. Taken together, these results suggest that shape and font qualities have differential influences on preferences and processing of objects and words.

## 1. Introduction

Humans are highly efficient in recognizing many common categories, including objects and words, regardless of a wide range of variations in visual qualities, such as styles and designs. Although such visual qualities may not affect recognition of an object or word (e.g., an office chair and a designer chair are both chairs, and 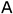 and 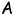 represent the same letter), these seemingly low-level visual properties are rapidly extracted to shape our preference towards the item. Preference judgments are often made quickly and without reasoning [1,2]. Such initial evaluations (e.g., likeability, preference) may determine our actions (e.g., to approach or avoid), and are made for almost everything that we encounter, including objects with either strong positive or negative associations (e.g., a flower, [3,4]) or relatively neutral associations (e.g., a teapot, [5]).

Although preferences are subjective, previous research has shown that preference judgments for visual qualities of objects and words are highly consistent among observers despite some individual differences ([2,6]). For both categories, preferences for curvy contour or roundness have been reported. For either familiar objects (e.g., sofa) or unfamiliar objects (e.g., abstract shapes or patterns), those with curvy shapes are consistently preferred compared with those with angular shapes (e.g., [7,8,9]). Likewise, rounder fonts are also consistently preferred over angular fonts (e.g., [10]). Remarkably, the preference for curvy rather than angular items does not appear to change when observers only see the images briefly (e.g., 84–300 ms, [2]) or until a response (e.g., [11]), and can be found across various tasks (e.g., making a binary like/dislike judgment, e.g., [7,11]; select a preferred item from a pair, or select a score on a preference rating scale, [11]). Although similar preferences for specific kinds of visual qualities, particularly curvy contour or roundness, have been observed for various categories, it remains unclear whether such visual qualities share similar processing mechanisms or have similar functions for the respective categories.

It is possible that similar mechanisms may underline the preferences for curvy shapes and fonts. Preference is suggested to be related to factors such as an aesthetic preference for curvy shapes as they are visually pleasant [8,12,13] or a sense of potential threat implied by angular shapes [14,15]. Although shapes and fonts may be regarded as low-level visual qualities of objects and words, shapes and fonts appear to carry meaning over-and-above the actual semantic meaning of the objects or words. For instance, simple curvy and angular shapes are associated with different kinds of meaning. Specifically, curvy shapes are associated with words that are related to safety (e.g., comfort, home) or positivity (e.g., laughter, success) and female names, whereas angular shapes are associated with words that are related to danger (e.g., bomb, torture) or negativity (e.g., rejected, evil) and male names [9]. Likewise, fonts appear to generate their own associations of meaning independent of the word they were used to write [10]. For instance, italicized fonts are generally perceived to be more active but less potent than regular Roman fonts [16], and 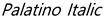 was associated with a connotation of “fast” whereas 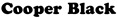 was associated with being “slow” [17]. Moreover, font qualities influence categorization judgments of category membership (e.g., whether “robin” or “penguin” are “birds”, [18]) and perceived psychological distance (e.g., a disfluent font renders the item more abstract and farther away, [19]). There are even reports of cross-modal correspondences between visual qualities and tastes, in which round shapes and fonts are associated with sweetness and angular shapes and fonts are associated with bitterness [10,20,21].

Preference is also thought to be related to perceptual fluency [22], as revealed by fast response times. Indeed, when comparing the processing of curvy and angular shapes that are comparable with objective complexity, curvy shapes were processed faster than angular shapes across multiple tasks, including categorization, same-different judgment, rotation, and symmetry detection [8,23]. Similarly, a familiar and clearly readable font results in faster and better identification, though not detection, of words [22]. The fluency and faster processing speed for curvy over angular shapes and fonts might be related to learned statistical regularities that are utilized for categorization in real-world experience. For instance, animals are recognized faster than man-made objects, perhaps partially due to the mid-level visual differences among the two categories: animals tend to have curvy or round shapes, whereas man-made objects are often boxy or elongated ([24,25]; see also [26]). Nonetheless, as comparable response times between curvy and angular objects were also sometimes observed for preference judgment, it has been suggested that the preference for curvy over angular shapes might also arise beyond perceptual fluency [7]. 

As the qualities of shapes and fonts show similar characteristics with regard to aesthetic impressions, semantic associations, and perceptual fluency, the present study examined how these qualities may contribute to preferences for object-word pairs. This question is theoretically important because although preference judgments can be made for both shape and font qualities of objects and words, it remains unclear whether the same factors underlie the influences of these visual qualities on the processing of objects and words. New insights on the roles of preferred or disliked shape and font qualities on the evaluation process can be revealed and compared when the two categories are examined directly. Apart from preference ratings, additional measures such as response times and eye movements during the evaluation of the object-word pairs allow for the understanding of the impact of shape and font qualities on processing time of and attention allocation towards objects and words. Note that preference ratings and response times could reveal similar or different patterns of results, which would suggest whether or how subjective preference and processing fluency for visual qualities of objects and words might be related, and how attention might be allocated to the object or the word depending on the visual qualities. Moreover, although objects and words are often studied separately in a laboratory setting, it is quite common that object-word pairs are shown simultaneously as a unit in everyday life, such as in the case of logos. Therefore, an investigation on shape and font qualities of object-word pairs may also have real-world implications on preferences for visual design of new brand logos [27].

In this study, we manipulated the preferred vs. disliked qualities for both shapes and fonts for object-word pairs. To minimize the potential semantic associations of familiar objects and words on the perception of shapes and fonts (e.g., [17]), only abstract objects and pseudowords were used in this study. If observers evaluate information from both objects and words equally, and the effects of preferred vs. disliked visual qualities from both categories are addictive, the highest preference ratings would be expected for object-word pairs with preferred shapes and fonts, whereas the lowest preference ratings would be expected for the pairs with disliked shapes and fonts. Another possibility is that the congruency between the shape and font qualities of the object-word pairs is also evaluated (e.g., see [28]), thus higher preference ratings would be expected for object-word pairs with preferred qualities for both components and for pairs with disliked qualities for both components, compared with pairs with preferred qualities for one component but disliked qualities for the other component. Alternatively, preferences and response times for object-word pairs might instead be driven by the visual qualities of only one but not both components, presumably the first component that observers attend to.

To examine how attention might be allocated to either the object or the word with different visual qualities, eye movements were recorded during the preference judgment. We focused on the measures of first fixations and total fixations towards the object-word pairs. Previous studies showed that observers’ first fixations and total fixation durations for pictorial or textual components depend on the relevance to their goals [29]: observers generally attended to the text over the image quicker and for a longer time on printed or webpage advertisement when considering making a purchase [30,31], but attended to the image quicker and for a longer time, compared with the text, when judging how much they liked the ad or how effective the ad was [32]. Based on these findings, we expected that when making the preference judgment of object-word pairs, the shape qualities might have a larger impact than font qualities, since the object might be fixated first and for a longer time, compared with the word. Moreover, differences in eye movements might also be expected between preferred and disliked qualities of shapes and fonts, since observers might be more likely to attend to preferred shapes and avoid attending to disliked shapes [8,33], but might be less likely to attend to preferred than disliked fonts [34,35,36]. 

## 2. Materials and Methods

### 2.1. Participants

Twenty-four undergraduate students (8 males and 16 females, *M* age = 19.42, *SD* = 1.39) from New York University Abu Dhabi (NYUAD) participated for course credits or subsistence allowance. All participants reported normal or corrected-to-normal vision. The study was approved by the NYUAD Institutional Review Board. All participants gave informed written consent prior to the experiment.

### 2.2. Apparatus

The stimuli were presented on a BenQ XL2411Z monitor using a Windows desktop computer and participants responded by pressing one of the dedicated keys on a standard keyboard. Eye movements were measured using an EyeLink 1000 Plus eye tracker (SR Research Ltd., Ottawa, ON, Canada) operating at 1000 Hz. Head movement was stabilized using a chin and head rest.

### 2.3. Stimuli and Procedure

#### 2.3.1. Pilot Study

We conducted a pilot study to select the shapes and fonts for the main study. In the pilot study, a separate group of 18 participants (10 males and 8 females, *M* age = 22.67, *SD* = 6.32) from the NYUAD community were presented with 400 grayscale images and were asked to provide preference ratings. Of those images, there were 200 different abstract shapes and the same letter-string ‘ABCDE’ writing in 200 different fonts. All of the abstract shapes were selected from the image set in [7], with 100 curvy shapes and 100 angular shapes. The 200 fonts were selected from a wide range of designs. Each shape or letter string was presented on a gray background of 256 pixels by 256 pixels in size. The presentation order of shape and font trials was blocked and counterbalanced across participants. On each trial, participants were first shown a fixation for 600 ms and then an image for 400 ms and were asked the extent they liked each shape or font on a 5-point scale, with 1 being the lowest (‘not at all’) and 5 being the highest (‘very much’). Participants were asked to respond as soon as possible, or within 10 s after stimulus onset (the average response time was 1302 ms, *SD* = 958 ms).

For the analysis of the pilot results, the trials with very brief response times were excluded (<300 ms, 194 out of 7200 trials = 2.69%). According to the ratings collected in the pilot study, we selected a total of 96 shapes and 96 fonts. For both categories, we selected 48 images with high ratings and 48 images with low ratings. Note that for the shapes, we selected from only the curvy shapes for the preferred condition and only the angular shapes for the disliked condition. Two-sample *t*-tests confirmed that the preferred shapes (*M* = 3.02, *SD* = 0.30) were rated significantly higher than the disliked shapes (*M* = 2.37, *SD* = 0.27), *t*_94_ = −11.23, *p* < 0.0001, *d* = −2.29 and the preferred fonts (*M* = 3.24, *SD* = 0.21) were rated significantly higher than the disliked fonts (*M* = 2.03, *SD* = 0.11), *t*_94_ = −35.91, *p* < 0.0001, *d* = −7.33. There was also no significant difference in response times either between preferred (*M* = 1229 ms, *SD* = 101 ms) and disliked (*M* = 1232 ms, *SD* = 142 ms) shapes, *t*_94_ = 0.10, *p* = 0.92, *d* = 0.02, or between preferred (*M* = 1317 ms, *SD* = 208 ms) and disliked (*M* = 1346 ms, *SD* = 211 ms) fonts, *t*_94_ = 0.68, *p* = 0.50, *d* = 0.14.

#### 2.3.2. Main Study

**Figure 1:**
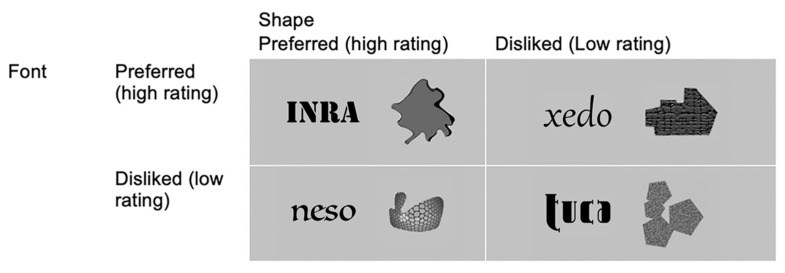
Sample object–word stimuli in the four main experimental conditions: preferred shapes with preferred and disliked fonts (**top-left** and **bottom-left**) and disliked shapes with preferred and disliked fonts (**top-right** and **bottom right**).

In the main study, each stimulus was composed of one of the 96 shapes (48 preferred and 48 disliked shapes) and a pseudoword in the English language in one of the 96 fonts (48 preferred and 48 disliked fonts), presented side-by-side, with either the shape or the word presented immediately left or right of the center of the screen. Figure 1 illustrates the sample stimuli. There were a total of 48 four-letter nonsense words (e.g., ‘dipo’, ‘lomu’), each word presented once in a preferred font and once in a disliked font for each participant. We made two sets of pairings between each pseudoword and a preferred/disliked font to be presented to different participants (e.g., ‘dipo’ was paired with two different preferred fonts and two different disliked fonts), with the two sets counterbalanced across participants to minimize any possible effects that the preference judgment was made based on the pseudowords but not the fonts.

Each shape or font was only shown once in the experiment to minimize prior exposure that could affect preference judgment [37]. The shapes and fonts were paired randomly on each trial. Specifically, half of the preferred shapes were paired with preferred fonts, whereas the rest of the preferred shapes were paired with disliked fonts. Likewise, half of the disliked shapes were paired with preferred fonts, whereas the rest of the disliked shapes were paired with disliked fonts. With each of the 4 shape–font conditions, the positions of the objects and words were counterbalanced: half of the trials showed the objects on the left and the words on the right, and the rest of the trials showed the words on the left and the objects on the right. There were a total of 192 trials, with 24 trials in each of the Shape quality (preferred vs. disliked), Font quality (preferred vs. disliked) and Position (shape-on-the-left vs. shape-on-the-right) conditions. Each object–word pair was presented on a gray background of 512 pixels in width and 256 pixels in height, subtending a visual angle of 11.35° in width and 5.68° in height. The analysis of eye movement data towards the objects and words excluded the gap between the two stimuli (80 pixels).

Participants were asked to treat each object–word pair as a logo and to indicate their preference for each logo on a 5-point scale (1 indicates ‘not at all’, whereas 5 indicates ‘very much’). On each trial, a fixation was presented at the center of the screen for 600 ms, followed by an object–word pair for 2 s. Participants were told to look at each display until the stimulus disappeared but were allowed to respond as soon as they saw the display, or up to 10 s after the stimulus onset.

## 3. Results

**Figure 2:**
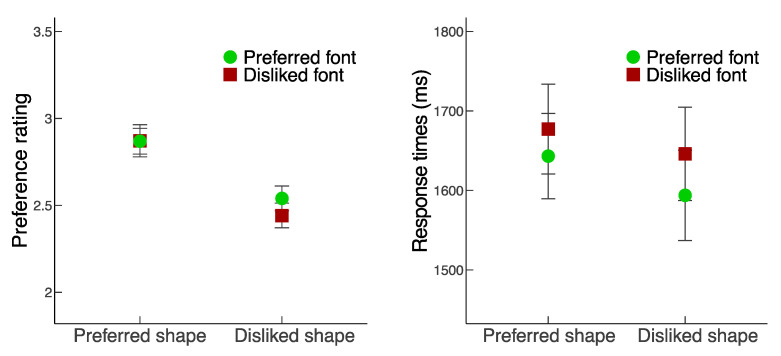
Preference ratings (**left**) and response times (RT, **right**) as a function of Shape quality and Font quality. Because the main effects and interactions involving the factor Position (i.e., whether the object or word was shown on the left vs. right) was not significant, the plotted results were collapsed for that factor. Error bars represent standard errors.

**Figure 3:**
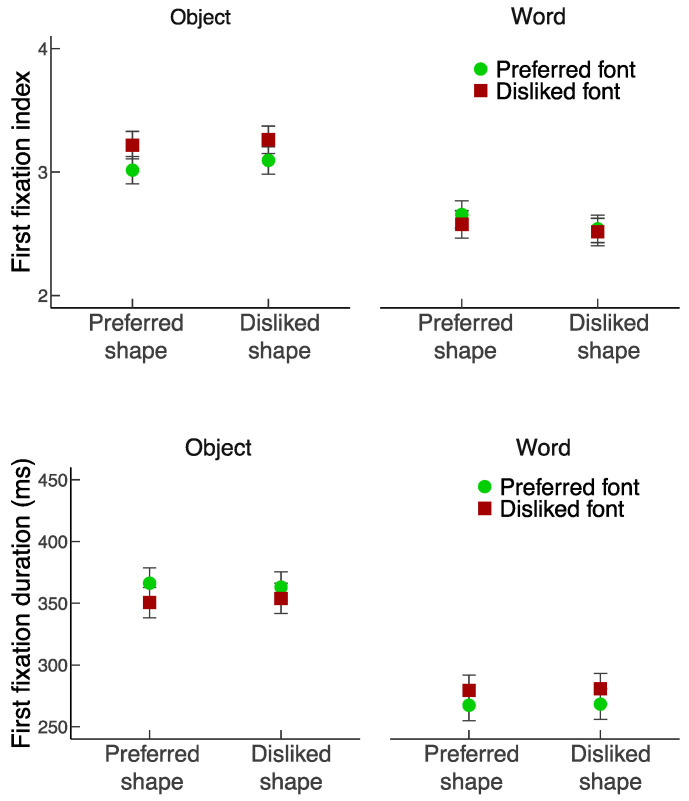
First fixation index (**top panel**) and first fixation duration (**bottom panel**) as a function of Shape quality and Font quality. Error bars represent standard errors.

**Figure 4:**
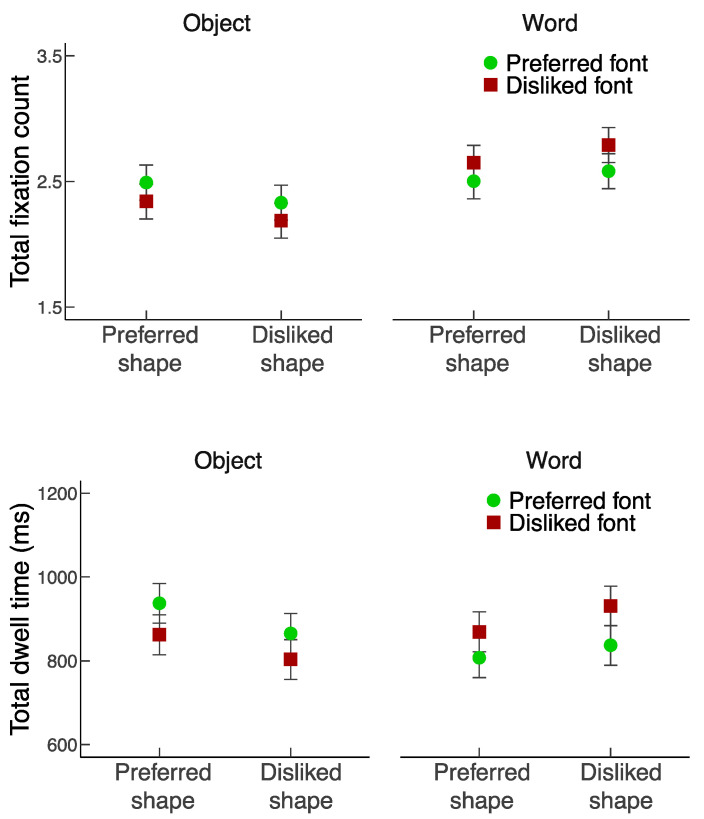
Total fixation count (**top panel**) and total dwell time (**bottom panel**) as a function of Shape quality and Font quality. Error bars represent standard errors.

For the analyses of preference ratings and response times, trials with extremely short (<200 ms) or extremely long (>4000 ms) response times were excluded (1.7% of the total number of trials). For the eye movement analysis, fixations that coincided with stimulus onset or the response, or extreme outliers (<60 ms or >1200 ms fixation duration) were excluded (4.6% of the total number of trials), resulting in a final data set consisting of 36,046 (out of 37,784) fixations. Figure 2 illustrates the preference ratings and response time results. Figure 3 and Figure 4 illustrate the results regarding the first fixations and the total fixations. 

### 3.1. Preference Ratings

An ANOVA was conducted on preference ratings with three within-subject factors: Shape quality (preferred vs. disliked), Font quality (preferred vs. disliked), and Position (left vs. right of center of screen). There was a significant main effect of Shape quality, *F*_1,23_ = 39.68, *p* < 0.001, *η_p_*^2^ = 0.633, with higher ratings for preferred than disliked shapes. Both the main effects of Font quality (*F*_1,23_ = 0.68, *p* = 0.417, *η_p_*^2^ = 0.029) and Position (*F*_1,23_ = 0.47, *p* = 0.502, *η_p_*^2^ = 0.02) were not significant. There were also no significant two-way (Shape quality × Font quality: *F*_1,23_ = 2.05, *p* = 0.165, *η_p_*^2^ = 0.082; Shape quality × Position: *F*_1,23_ = 0.46, *p* = 0.506, *η_p_*^2^ = 0.019; Font quality × Position: *F*_1,23_ = 0.09, *p* = 0.765, *η_p_*^2^ = 0.004) or three-way interactions (*F*_1,23_ = 0.19, *p* = 0.666, *η_p_*^2^ = 0.008). These results suggest that regardless of the positions of the objects or words, participants relied on the shape quality of the object instead of the font quality of the word for preference judgment. 

### 3.2. Response Times (RT)

An ANOVA was conducted on RT with three within-subject factors: Shape quality (preferred vs. disliked), Font quality (preferred vs. disliked), and Position (left vs. right of center of screen). There was a main effect of Shape quality, *F*_1,23_ = 5.00, *p* = 0.035, *η_p_*^2^ = 0.18, with slower responses for preferred shapes than disliked shapes. There was also a main effect of Font quality, *F*_1,23_ = 5.61, *p* = 0.027, *η_p_*^2^ = 0.20), with faster responses for preferred fonts than disliked fonts. The main effect of Position was not significant, *F*_1,23_ = 1.80, *p* = 0.19, *η_p_*^2^ = 0.072. There were no significant two-way interactions (Shape quality × Font quality: *F*_1,23_ = 0.27, *p* = 0.61, *η_p_*^2^ = 0.012; Shape quality × Position: *F*_1,23_ = 0.1, *p* = 0.749, *η_p_*^2^ = 0.005; Font quality × Position: *F*_1,23_ = 2.24, *p* = 0.148, *η_p_*^2^ = 0.089) or three-way interactions (*F*_1,23_ = 3.46, *p* = 0.076, *η_p_*^2^ = 0.13). 

### 3.3. Eye Movements

For the eye movement data, we focused on four measures on objects and words separately: first fixation index, first fixation duration, total fixation count, and total dwell time. First fixation index on either the object or the word indicated the number of fixations it took the participant to first fixate each component. First fixation duration indicated the duration of the first fixation on each component. Total fixation count on either the object or the word indicated the total numbers of fixations made on each component. Total dwell time indicated the duration of all fixations on each component. A three-way ANOVA was conducted on each of these measures with within-subject factors: Focus (object vs. word), Shape quality (preferred vs. disliked), and Font quality (preferred vs. disliked). The analysis was conducted on data collapsed across the left vs. right positions for the objects and words, because the analysis focus was on the fixations to the categories (object vs. word), and the factor Position was counterbalanced across conditions. 

#### 3.3.1. First Fixation Index

There was a main effect of Focus, *F*_1,23_ = 17.62, *p* < 0.001, *η_p_*^2^ = 0.434, with the first fixations made more frequently to the word than the object. The main effect of Shape quality was not significant, *F*_1,23_ = 0.06, *p* = 0.801, *η_p_*^2^ = 0.003. There was a main effect of Font quality, *F*_1,23_ = 4.36, *p* = 0.048, *η_p_*^2^ = 0.159, which was modulated by a significant interaction between Focus and Font quality, *F*_1,23_ = 7.67, *p* = 0.011, *η_p_*^2^ = 0.25, with the effect of Font quality observed when the Focus was on the object but not the word. Specifically, although the first fixations were more likely made on the word than the object in all conditions (*p*’s < 0.02, Scheffé’s test), the difference was larger with the presence of disliked than preferred fonts (*p* < 0.01), suggesting that when the word was shown in disliked rather than preferred fonts, participants took additional fixations on the word prior to initiating a fixation to the object, presumably because disliked fonts required further processing than preferred fonts. There were no other significant two-way interactions (Focus × Shape quality: *F*_1,23_ = 2.79, *p* = 0.109, *η_p_*^2^ = 0.108; Shape quality × Font quality: *F*_1,23_ = 0.01, *p* = 0.914, *η_p_*^2^ < 0.001) or three-way interaction (*F*_1,23_ = 0.33, *p* = 0.572, *η_p_*^2^ = 0.014).

#### 3.3.2. First Fixation Duration

There was a main effect of Focus, *F*_1,23_ = 76.38, *p* < 0.001, *η_p_*^2^ = 0.769, with longer first fixations on objects than words. There were no main effects of either Shape quality (*F*_1,23_ = 0.02, *p* = 0.88, *η_p_*^2^ = 0.001) or Font quality (*F*_1,23_ < 0.01, *p* = 0.99, *η_p_*^2^ < 0.001). There was a significant interaction between Focus and Font quality, *F*_1,23_ = 11.96, *p* = 0.002, *η_p_*^2^ = 0.342: although the first fixations were longer for objects than words in all conditions (*p*’s < 0.001, Scheffé’s test), the difference was reduced with the presence of disliked than preferred fonts (*p* = 0.002), presumably because participants switched attention from the object back to the word faster for disliked fonts, which likely required further processing, than for preferred fonts (*p* < 0.001, Scheffé’s test). There were no other significant two-way interactions (Focus × Shape quality: *F*_1,23_ = 0.02, *p* = 0.897, *η_p_*^2^ < 0.001; Shape quality × Font quality: *F*_1,23_ = 0.53, *p* = 0.474, *η_p_*^2^ = 0.023) or three-way interaction (*F*_1,23_ = 0.02, *p* = 0.622, *η_p_*^2^ = 0.008).

#### 3.3.3. Total Fixation Count

There were no significant main effects of Focus (*F*_1,23_ = 2.77, *p* = 0.109, *η_p_*^2^ = 0.108), Shape quality (*F*_1,23_ = 0.89, *p* = 0.355, *η_p_*^2^ = 0.037), nor Font quality (*F*_1,23_ = 0.52, *p* = 0.478, *η_p_*^2^ = 0.022) on total fixation count. However, there was an interaction between Focus and Shape quality, *F*_1,23_ = 20.9, *p* < 0.001, *η_p_*^2^ = 0.476, and an interaction between Focus and Font quality, *F*_1,23_ = 26.47, *p* < 0.001, *η_p_*^2^ = 0.535. Essentially, participants made approximately five fixations across each object–word pair. Although the fixations were quite evenly distributed across the object and the word, participants fixated more frequently the word with disliked than preferred fonts (*p* = 0.007, Scheffé’s test), and on the object with preferred than disliked shapes (*p* = 0.006, Scheffé’s test). The interaction between Shape quality and Font quality (*F*_1,23_ = 0.55, *p* = 0.465, *η_p_*^2^ = 0.023) and the three-way interaction (*F*_1,23_ = 0.21, *p* = 0.654, *η_p_*^2^ = 0.009) were not significant.

#### 3.3.4. Total Dwell Time

The results of total dwell time were highly similar to those of total fixation count. There were no significant main effects of Focus (*F*_1,23_ = 0.01, *p* = 0.939, *η_p_*^2^ < 0.001), Shape quality (*F*_1,23_ = 1.07, *p* = 0.312, *η_p_*^2^ = 0.044), nor Font quality (*F*_1,23_ = 0.32, *p* = 0.574, *η_p_*^2^ = 0.014) on total dwell time. However, there was an interaction between Focus and Shape quality, *F*_1,23_ = 19.69, *p* < 0.001, *η_p_*^2^ = 0.461, and an interaction between Focus and Font quality, *F*_1,23_ = 37.95, *p* < 0.001, *η_p_*^2^ = 0.623. Similar to the total fixation count results, although the total dwell time on the object and the word was comparable, participants looked longer at the word with disliked than preferred fonts (*p* = 0.001, Scheffé’s test), and at the object with preferred than disliked shapes (*p =* 0.0005, Scheffé’s test). The interaction between Shape quality and Font quality (*F*_1,23_ = 2.03, *p* = 0.168, *η_p_*^2^ = 0.081) and the three-way interaction (*F*_1,23_ = 0.25, *p* = 0.624, *η_p_*^2^ = 0.011) were not significant.

## 4. Discussion

To examine the effects of visual qualities on the evaluation of object-word pairs, we manipulated the qualities of shapes and fonts. We measured subjective preference, response times, and eye movements during the evaluation and observed different patterns of results across the measures. 

We found that preference ratings for the object-word pairs were primarily determined by the shape qualities of the objects, whereas the font qualities of the words appeared to have negligible effects. Specifically, higher preference ratings were observed when the objects had preferred than disliked shapes, regardless of whether the words were shown in preferred or disliked fonts. Moreover, the congruency between shape and font qualities did not appear to influence the preference ratings for the object-word pairs. This result might be surprising: although both the preferred and disliked qualities of the shapes and fonts had been established in the pilot study, participants only relied on the shape qualities even when both sets of information were available in the main study. Consistent with previous studies, it is possible that during object processing, curvy shapes are generally preferred because of an aesthetic pleasantness [8,12,13] and angular shapes are generally disliked because of an indication of potential threat [14,15]. On the other hand, aesthetic values of font qualities may not be as critical during word processing even though preference judgment can be made for fonts.

Nonetheless, both shape and font qualities had an impact on the evaluation process, as the response times for the preference decisions were influenced by both sets of qualities. More specifically, preferred vs. disliked shape and font qualities showed different effects, with longer response times found for preferred than disliked shapes and for disliked than preferred fonts. Although no differences in response times were observed for either shapes or fonts during the pilot study, it is important to emphasize that because opposite patterns of response times and total fixations were observed for preferred vs. disliked shape and font qualities, it is unlikely that the same factors affect the processing of objects and words equally. Presumably, preferred shapes are more pleasant to look at compared with disliked shapes [8,12,13,33], while it may take longer to identify the words shown in disliked than preferred fonts (e.g., [10]). Similar differential effects of preferred vs. disliked shape and font qualities were also revealed in two of the eye movement measures, specifically the total fixation count and total dwell time. Taken together, these results suggest that shape and font qualities do not produce identical effects on either preference ratings or processing time, and the amount of processing time for specific shape and font qualities is unlikely related to subjective preference for object-word pairs.

Although participants evaluated both components of the object-word pairs, as they made comparable numbers of fixations to each component and fixated each component for approximately the same amount of time, subjective preference for object-word pairs may instead depend on certain aspects of the first fixations that participants made to either the object or the word. Since preference judgment is often made rapidly [2,7], it is conceivable that the first impression for a single component could determine the preference towards the pair. We measured whether participants attended to the object or the word first (first fixation index) and how long the first fixation was (first fixation duration). These two measures revealed the opposite results: participants attended to the word before the object, but the first fixation on the object was longer than the first fixation on the word. Such results were only partially consistent with previous findings of stronger influences of the pictorial than textual components during preference judgment, as the pictorial component of a printed advertisement was both fixated quicker and longer than the textual component [32]. Nonetheless, because the preference ratings for object-word pairs were found to depend on only the shape qualities of the object and not the font qualities of the word, our results suggest that the preference judgment was not necessarily based on the component that participants first attended to (i.e., the word), but instead the first component that they examined for a sufficient amount of time (i.e., the object).

Why do words attract attention first and why do shape qualities of an object show stronger influences on preference judgment than font qualities of a word? We suggest that the different processing goals for the two categories, or the differential functionality of the visual qualities of each of the categories, may play an important role. It is possible that attention is first attracted to words than objects because it is quicker to process word meaning than shape meaning. Indeed, faster responses were found in a congruence judgment task when observers attended to the word before the object in object-word pairs (e.g., both are squares/circles, or one is a square and the other is a circle) [38]. More importantly, the impact of visual qualities may depend on the processing needs required for extracting meaning from the specific categories. For object processing, the shape qualities may signify the visual appearance but not necessarily the essence of the object (e.g., different shapes of sofas). In contrast, for word processing, the legibility of the font is critical to identify the letters and access the word meaning and disliked fonts are often considered less legible than preferred fonts [10]. Therefore, although the qualities of shapes and fonts can both be evaluated and the preferred qualities of shapes and fonts are appreciated, the desirable characteristics of shapes and fonts serve different processing goals for extracting meaning from the categories.

As our study aimed to examine the relative importance of shape and font qualities on preference judgment on object-word pairs, we expected that the participants would attend to either or both components in a serial manner. However, it is possible that participants might instead fixate a central point of the display to evaluate the pair as a unit. Because our stimuli were presented at the center of the screen, where participants fixated prior to the stimulus onset, we were not able to distinguish whether any initial fixations located at the center of the display were relevant to the preference judgment. To clarify the eye movement strategies used by observers when viewing the object-word pairs, future studies may consider presenting the displays off center and examining whether participants adopt a holistic or analytical viewing strategy (e.g., [39]). Furthermore, since shapes and fonts alone may convey meaning [9,17], future investigation on shape and font qualities could use familiar objects and meaningful words to examine whether the effect of shape qualities on preference judgment remains robust, or whether higher-level factors such as semantic processing would instead lead to a stronger emphasis on font qualities. While our findings suggest that shape qualities have a more critical role than font qualities on subjective preference, further studies may examine the real-world implications of shape and font qualities on visual design of familiar compared with novel brand logos.

## 5. Conclusions

Although preferred and disliked shape and font qualities can be consistently identified among observers, the present study revealed that subjective preference for object-word pairs was determined primarily by shape qualities of the object but not font qualities of the word. By studying the visual qualities of objects and words together, our findings provide new insights that the desirable shape and font qualities may serve different processing goals for the categories.

## Data Availability

The data and analysis code for this study are available at https://osf.io/56h8s/.
